# Splenic Artery Embolization for Upper Gastrointestinal Bleeding Caused by Hemosuccus Pancreaticus

**DOI:** 10.1155/crgm/6149221

**Published:** 2025-02-20

**Authors:** Evghenii Gutu, Dumitru Casian, Roman Smolnitchi, Vasile Culiuc, Andrei Scureac

**Affiliations:** ^1^Department of General Surgery, “Nicolae Testemitanu” State University of Medicine and Pharmacy, Chisinau, Moldova; ^2^Department of Surgery, Division of Vascular Surgery, Institute of Emergency Medicine, Chisinau, Moldova

**Keywords:** chronic pancreatitis, gastrointestinal bleeding, hemosuccus pancreaticus, splenic artery pseudoaneurysm, transcatheter coil embolization

## Abstract

Hemosuccus pancreaticus (HP) is a rare but potentially life-threatening condition, characterized by upper gastrointestinal bleeding from the ampulla of Vater, often originating from a ruptured pseudoaneurysm of the peripancreatic arteries. Despite its rarity, HP presents a diagnostic and therapeutic challenge due to its elusive clinical presentation and complex underlying pathophysiology. In this case report, we presented a compelling instance of HP, diagnosed in a 48-year-old man, complicated with gastrointestinal bleeding and severe anemia successfully managed with urgent endovascular intervention. We highlighted the importance of early recognition, prompt intervention, and interdisciplinary collaboration in achieving favorable outcomes in patients afflicted by this distinctly unusual condition.

## 1. Introduction

Hemosuccus pancreaticus (HP), an extremely rare but potentially life-threatening condition, is characterized by bleeding into the pancreatic duct, often originating from a ruptured pseudoaneurysm of the peripancreatic arteries [1, 2]. Despite its rarity, HP presents a diagnostic and therapeutic challenge due to its elusive clinical presentation and complex underlying pathophysiology.

Endovascular interventions have emerged as a promising approach in the management of HP, offering a minimally invasive alternative to open surgery [3]. These techniques encompass a spectrum of procedures including selective diagnostic angiography, coil embolization, and stent placement, tailored to the specific vascular anatomy and pathology contributing to the bleeding.

In this case report, we presented a compelling instance of HP complicated with gastrointestinal bleeding and severe anemia successfully managed with urgent endovascular intervention. Through a comprehensive review of the patient's clinical course, diagnostic workup, and therapeutic strategy, we aimed to shed light on the efficacy and feasibility of endovascular approaches in the treatment of this rare vascular complication of chronic pancreatitis. By documenting this case and discussing available literature, we underscored the importance of early recognition, prompt intervention, and interdisciplinary collaboration in achieving favorable outcomes in patients afflicted by this distinctly unusual condition.

## 2. Case Presentation

A 48-year-old man was admitted to the Department of Internal Medicine with severe anemia. He reported weakness, dizziness, and intermittent abdominal pain in the epigastric region for about 2 weeks. The patient denied any obvious signs of blood loss such as bloody diarrhea, melena, coffee ground emesis, hemoptysis, or hematuria. His past medical history was remarkable for chronic pancreatitis, long-term alcohol abuse and smoking. Several years ago, he had undergone median laparotomy for a penetrating abdominal knife wound with a small bowel injury. At presentation, the patient appeared pale but had relatively stable hemodynamic parameters with blood pressure of 110/70 mm·Hg, heart rate of 92 beats/min, and respiratory rate of 16 breaths/min. Physical examination revealed mild mid-epigastric tenderness on palpation, without rigidity and peritoneal signs. Laboratory tests showed severe anemia with a hemoglobin level of 4.7 g/dL, without any other abnormalities in total blood count and biochemistry, including serum amylase, lipase, and liver function tests. The patient received four units of packed red blood cells with an increase in hemoglobin to 8.3 g/dL and was then referred to the surgical department.

**Figure 1:**
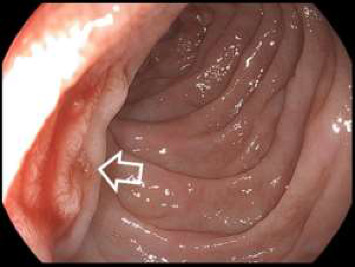
Upper gastrointestinal endoscopy revealing fresh blood oozing from the papilla of Vater (arrow).

**Figure 2:**
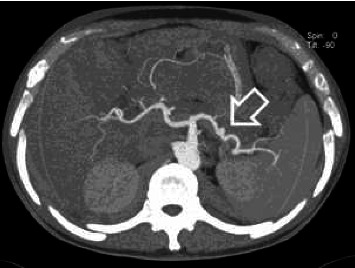
Axial computed tomography angiography of the abdomen shows a saccular pseudoaneurysm of the proximal splenic artery, measuring 6 mm (arrow).

**Figure 3:**
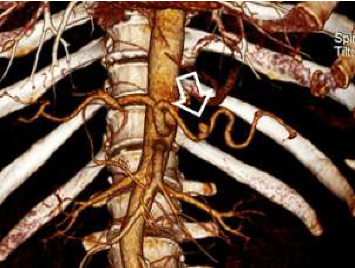
Multiplanar CT reconstruction demonstrating the pseudoaneurysm of the splenic artery (arrow).

**Figure 4:**
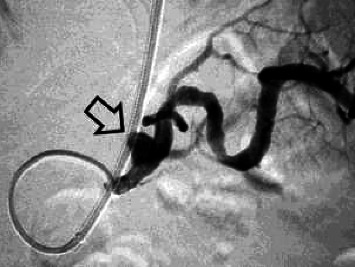
Selective angiography demonstrates a pseudoaneurysm of the splenic artery (arrow), without evident contrast extravasation into the main pancreatic duct.

**Figure 5:**
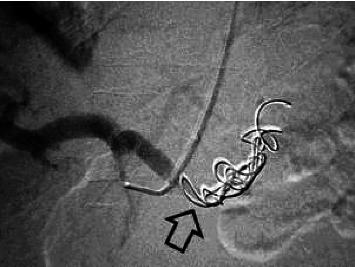
Postprocedural angiography demonstrating occlusion of the splenic artery with coils and exclusion of the pseudoaneurysm (arrow).

Abdominal sonography was inconclusive although suggested diffuse pancreatic parenchymal changes and hepatic steatosis. Upper gastrointestinal endoscopy revealed a significant amount of fresh blood in the second portion of the duodenum, and continuous blood oozing out of the major papilla (Figure 1). The subsequent abdominal contrast-enhanced computed tomography (CT) noted chronic pancreatitis with multiple small calcifications, mainly in the head of the gland, as well as a small (6 mm) saccular pseudoaneurysm of the splenic artery located close (22 mm) to its origin from the celiac trunk (Figure 2 and 3). Thus, the patient was diagnosed with HP, and urgent endovascular treatment was considered due to the ongoing bleeding and severe anemia. Under local anesthesia and mild sedation, the left brachial artery was punctured, and a guiding sheath was advanced in the proximal abdominal aorta, and then into the celiac trunk. Selective digital subtraction angiography confirmed the presence of the saccular splenic artery pseudoaneurysm, without visible extravasation of the contrast and communication with the Wirsung duct (Figure 4). The 0.035 guidewire (“Glidewire”, Terumo, USA) was placed into the distal splenic artery. The initial attempts to exclude pseudoaneurysm by implantation of a covered stent, preserving adequate arterial flow to the spleen, failed due to the impossibility of advancing the 5.0 × 28-mm stent-graft (“BeGraft”, Bentley InnoMed GmbH, Germany) through the sharply angulated segment between the celiac trunk and splenic artery. As a result, the plan of endovascular intervention was modified, and the patient underwent an uneventful coil embolization using two detachable helical hydrocoils 10 mm × 20 cm (“Azur-35” and “Azur-18”, Terumo, USA). Postembolization angiogram confirmed the complete luminal occlusion of the proximal splenic artery and ruled out any collateral filling of the pseudoaneurysm (Figure 5).

After the procedure, the patient was treated with intravenous hydration and proton-pump inhibitors, and liquid food was started on Day 2 while solid food—2 days later. His hemoglobin level remained stable and did not drop during the following days of hospitalization, maintaining above 8 g/dL. CT angiography confirmed successful coil embolization of the splenic artery with no splenic infarction. The patient was discharged home in satisfactory condition on Day 5 after the endovascular intervention. During the follow-up visits at 2 weeks, 1, 3, and 6 months, he did not develop any clinical symptoms or laboratory signs of recurrent gastrointestinal bleeding with gradual normalization of hemoglobin level.

## 3. Discussion

HP is a rare and potentially life-threatening clinical entity, characterized by upper gastrointestinal bleeding from the ampulla of Vater via the pancreatic duct [1, 2]. The first report of this condition belongs to Lower and Farrell, who in 1931 described bleeding from the upper digestive tract secondary to a splenic artery aneurysm [4]. Several terms have been proposed to define the bleeding from the pancreatic duct into the duodenum through the papilla, including “wirsungorrhagia,” “hemowirsungia,” “hemoductal pancreatitis,” “pseudohemobilia” [2, 5], and even anatomically more specific term, such as “santorinirrhage” [6]. However, the most accepted term has become “hemosuccus pancreaticus,” first introduced and popularized by Sandblom in 1970, who initially reported three patients with gastrointestinal bleeding secondary to the rupture of a pseudoaneurysm into the pancreatic duct [7].

Due to the extreme rarity of the condition, the medical literature on HP is mainly limited to case reports and small case series [8]. According to generally accepted opinion, only about 150 cases have been reported in the literature since the first case was published in 1931 [9, 10]. On the other hand, the incidence of HP is estimated to be one in 1500 patients presenting with upper gastrointestinal bleeding [1–3]. Although HP is uniformly considered as a rare condition, it remains a subject for discussion whether this is due to a true low incidence or rather a consequence of underdiagnosis [10].

Most commonly, HP develops secondary to the pathology of the pancreatic gland, mainly due to chronic pancreatitis and the formation of peripancreatic arteries pseudoaneurysm [5]. Other possible causes include acute pancreatitis, pancreatic pseudocyst, pancreatic tumors, pancreaticolithiasis, pancreas divisum, pancreatic trauma, or iatrogenic injuries—usually during the endoscopic retrograde cholangiopancreatography [3, 10, 11]. The mechanism of pseudoaneurysm formation implicates the release of pancreatic enzymes, resulting in lysis and gradual degradation of the arterial wall and surrounding pancreatic tissue [11, 12]. Erosion and rupture of pseudoaneurysm of the visceral artery into the pancreatic duct leads to a potentially life-threatening digestive hemorrhage [9]. The splenic artery is more vulnerable than other peripancreatic vessels, due to its long and tortuous course in proximity to pancreatic parenchyma [6]. Accordingly, pseudoaneurysm of the splenic artery represents the most common source of HP accounting for 38%–65% of the cases, followed by gastroduodenal, pancreaticoduodenal, hepatic, left gastric, and superior mesenteric arteries aneurysms [3, 13–15].

Despite the various possible etiologies, HP is most commonly diagnosed in young male patients with a history of chronic alcohol-related pancreatitis [6], as was mentioned in the presented clinical case. While there are no symptoms pathognomonic for HP, the presentation patterns that should raise the suspicion of this entity usually are described by a classical triad of gastrointestinal hemorrhage, epigastric abdominal pain, and hyperamylasaemia [9, 10]. The key symptom of upper gastrointestinal bleeding in the case of HP is melena, which is most common, whereas hematemesis is less frequent [6, 14]. The digestive hemorrhage usually is intermittent due to repeated formation and lysis of the clot in the pancreatic duct [9] and often not severe enough to cause hemodynamic instability. Occlusion of the pancreatic duct results in a temporary cessation of bleeding, followed by subsequent dissolution of blood clots and gradual decrease in pressure inside the pancreatic duct with the disappearance of abdominal pain [1, 15]. Elevated serum amylase level is a less constant sign from the “classical symptomatic triad” of HP and could be observed in a few patients only during the episodes of acute pancreatitis or exacerbation of chronic inflammation of the gland [5, 11].

HP frequently presents a diagnostic dilemma due to its rarity, variations in anatomical location and underlying pathophysiology, while intermittent signs and symptoms contribute to its difficult detection. Due to the lack of a standardized diagnostic approach, patients with HP usually undergo extensive workup before a diagnosis is made. As a rule, examination of patients with HP begins with upper endoscopy—an investigation focused on the main clinical manifestation of the disease, i.e., upper gastrointestinal bleeding. The active blood oozing from the ampulla of Vater represents a pathognomonic sign of HP; however, it can only be observed in a limited number of cases, ranging from 22% to 51% according to existing evidence [1, 5, 14]. Thus, initial endoscopic examination of the upper digestive tract frequently could offer negative results due to the intermittent nature of active bleeding [11, 14]. Nevertheless, even nondiagnostic digestive endoscopy is essential in ruling out other common causes of upper gastrointestinal bleeding such as peptic ulcer disease, erosive gastritis, esophageal varices, Dieulafoy's lesion, and gastrointestinal neoplasms [1]. Some studies suggest that the use of upper endoscopy with a side-viewing duodenoscope has a higher diagnostic capacity for intermittent bleeding from the major duodenal papilla [6], while subsequent endoscopic retrograde cholangiopancreatography could demonstrate a filling defect within the pancreatic duct caused by the presence of blood clots or compression with pseudoaneurysm [2, 3]. However, the use of cholangiopancreatography at an early stage of diagnostic workup must be justified by a strong suspicion of the diagnosis of HP. Moreover, it should be emphasized that all mentioned radiological findings are only suggestive of the diagnosis.

Contrast-enhanced CT scan is the preferred diagnostic tool, if there is suspicion of vascular complications and active bleeding due to underlying pancreatic pathologies, as in the case of HP. In addition, CT angiography provides a better understanding of the anatomical changes and is therefore of great importance in selecting further appropriate therapeutic interventions [13, 16]. Although the visualization of contrast leakage between a peripancreatic vessel and the pancreatic duct is extremely unusual, the finding of a visceral artery pseudoaneurysm in a patient with an unexplained source of upper gastrointestinal bleeding, especially in a patient with a pancreatic disease, although not definitive is certainly very suggestive of HP [12]. Furthermore, contrast-enhanced CT is an excellent modality for demonstrating the various pancreatic abnormalities: chronic pancreatitis, pancreaticolithiasis, pseudocysts, solid or cystic neoplasms, and neuroendocrine tumors. Overall, the sensitivity of CT for identification and localization of the source of bleeding in patients with HP is 90%–96% [6, 14], being relatively higher in cases with clinical evidence of severe bleeding and hemodynamic compromise.

Nowadays different strategies and modalities are available for the treatment of HP, all meeting the main goal—complete eradication of the source of bleeding. These include endovascular, percutaneous, and endoscopic ultrasound-guided and surgical treatment, whereas medical therapy seems to have no effect. The selection of certain options for endovascular treatment (coil embolization, balloon occlusion, and implantation of covered stent) depends on a variety of clinical, anatomical, technical, logistical, and other factors. The stenting procedure using a stent-graft is useful and recommended in patients with HP, when pseudoaneurysm is located proximally in a large vessel such as a hepatic, proximal splenic, or superior mesenteric artery to prevent embolism or ischemia to the major organs [13]. The stent excludes blood flow into the aneurysm preserving patency of the artery and maintaining adequate perfusion of the organs. A similar anatomical situation has been noted in the presented case, i.e., the pseudoaneurysm was located in the splenic artery close to its origin from the celiac trunk. However, advancing the stent to the arterial segment with pseudoaneurysm turned out to be technically impossible due to marked arterial angulation. Failure of stent grafting on itself is not surprising. In one recently published large series, stent implantation was successful in only one of 49 HP cases [6]. Therefore, the role of this newer modality as a therapeutic tool, as well as its technical aspects and limitations, needs to be determined in the future.

The endovascular occlusion of pseudoaneurysm with coils is an alternative curative approach in the case of HP [1, 11], although several other embolic agents such as n-butyl cyanoacrylate glue, gel-foam, thrombin, or vascular plugs can also be employed [12, 13]. During the embolization procedure, the pseudoaneurysm is filled with coils to stimulate thrombus formation in the sac of the aneurysm and occlusion of the artery. It is important to embolize both the proximal and distal ends of the artery to isolate the pseudoaneurysm from the blood supply and to achieve successful control of the bleeding [5]. Overall, this procedure is safe and efficient in hemodynamically stable patients. Published reports demonstrate a high success rate (75%–100%), low morbidity (14%–25%), and mortality (0%–33%) associated with transcatheter embolization [10, 13–15]. Moreover, several authors expect further improvement in the results of selective arterial embolization due to enhancement of operators' skills and advancement in angiographic equipment and techniques [3, 14].

Historically, surgery was considered the optimal treatment of HP since only resection can eliminate the pancreatic parenchyma involved in the pathological process and treat both the pancreatic and arterial diseases [12]. Additional arguments in favor of surgery appear if a patient has concomitant complications of chronic pancreatitis such as pseudocyst, benign biliary strictures, gastric outlet obstruction, painful inflammatory head mass, and suspicion of pancreatic tumor or intractable pain. Currently, surgical treatment is reserved for cases with massive hemorrhage and hemodynamic instability, when angiographic therapy is not available or fails, as well as for patients with ongoing or recurrent bleeding [2, 8]. Standard surgical procedures include intracystic arterial ligation, distant vessel ligation, distal pancreatectomy with or without splenectomy, pancreaticoduodenectomy, and pseudocyst drainage [8, 10–12]. The surgical treatment has a success rate of up to 70%–85% with low postoperative rebleeding rates, ranging from 0% to 5%. However, the operative mortality is estimated to be between 20% and 50%, being particularly high when the pseudoaneurysm is located in the head of the pancreas [5, 14].

In conclusion, HP is a rare but potentially life-threatening condition and should be included in differential diagnoses in patients with chronic pancreatitis presenting with acute or intermittent upper gastrointestinal bleeding. Although there are various diagnostic pathways in the case of HP, all of them should lead to early CT angiography, which is only enabling to confirm the diagnosis and establish the exact anatomy of the lesion. All hemodynamically stable patients with HP should undergo urgent endovascular treatment, the exact technique (stenting or embolization) being determined during the intervention. Indications for open surgery should be limited and reserved for cases with hemodynamic instability and ongoing bleeding or failed endovascular interventions.

## Data Availability

The data used to support this study are included within the article. Additional data are available from the corresponding author upon reasonable request.
